# Methamphetamine and Dopamine Receptor D1 Regulate Entrainment of Murine Circadian Oscillators

**DOI:** 10.1371/journal.pone.0062463

**Published:** 2013-04-23

**Authors:** Jennifer A. Mohawk, Pinar Pezuk, Michael Menaker

**Affiliations:** Department of Biology, University of Virginia, Charlottesville, Virginia, United States of America; Nagoya University, Japan

## Abstract

We investigated the effect of methamphetamine (MA) injections on the circadian organization of behavior and individual tissues in the mouse. Scheduled, daily injections of MA resulted in anticipatory activity, with an increase in locomotor activity immediately prior to the time of injection. Daily MA also shifted the peak time of PER2 expression in the liver, pituitary, and salivary glands. It has been suggested that reward pathways, and dopamine signaling in particular, may underlie the effects of MA on the circadian system. To test this hypothesis, we examined the effect of the D1 receptor antagonist SCH23390 (SCH) on circadian rhythms. The MA-induced shift in the phase of pituitary and salivary glands was attenuated by pretreatment with the D1 antagonist SCH23390 (SCH). Interestingly, daily SCH, administered alone, also affected some circadian oscillators. The livers and lungs (but not pituitaries or salivary glands) of mice treated with daily injections of SCH displayed disrupted rhythms of PER2 expression, suggesting that D1 receptor signaling is important for entrainment of these organs. From these results, we conclude that MA has widespread effects within the circadian system, and that these effects are mediated, at least in part, by the dopaminergic system. This study also identifies a role for dopamine signaling in normal entrainment of circadian oscillators.

## Introduction

Circadian rhythms, approximately 24h rhythms of behavioral and physiological processes, are regulated in mammals by a central pacemaker within the suprachiasmatic nucleus (SCN) of the hypothalamus [1], [2]. While light is normally the strongest zeitgeber (“time-giver”) for the circadian system, keeping organisms entrained to the external environment, it is clear that other factors, including food and drugs, are capable not only of entraining the circadian system, but also of driving rhythms in the absence of the SCN [3]–[5]. The stimulant drug of abuse methamphetamine has particularly profound effects on circadian rhythms [6]–[9], but the way in which this drug interacts with the SCN (and potentially other circadian oscillators) to affect the brain and peripheral organs is not well understood.

Scheduled, daily injections of methamphetamine (MA) result in increased activity levels following [8], [10]–[12], and in some cases prior to [8], [10], the time of expected drug delivery. This phenomenon has been documented in both rats and mice, and robust increases in activity levels can be observed following the expected time of injection even on a day when the drug is withheld [8], [13]. The MA-induced change in the normal circadian pattern of activity is associated with a phase advance in the expression of the clock genes *Per1* and *Per2* within the striatum but not the SCN [13]. Scheduled MA-injections given earlier in the light period have recently been shown to delay *Per2* rhythms in a variety of extra-SCN brain regions [11]. Scheduled injections of MA also reinstate behavioral circadian rhythms in otherwise arrhythmic SCN-lesioned animals [13]. These rhythms persist on withdrawal days and are associated with a reinstatement of circadian rhythms of *Per* expression in the striatum and liver of the SCN-lesioned animals [13]. This suggests that the activity and clock gene expression rhythms driven by scheduled MA injections are SCN-independent. Given the ability of scheduled MA injections to influence clock gene expression in the brain and liver, we hypothesized that daily injections of MA in SCN-intact animals would shift the phase of other circadian oscillators in individual organs without affecting rhythms in the SCN.

The effect of scheduled MA injections on circadian locomotor activity is, at least partly, due to activation of dopaminergic pathways. Both D1 and D2 dopamine receptor antagonists (as well as NMDA antagonists) are capable of blocking the increased activity observed on MA withdrawal days [12]. In this study we chose to focus specifically on a D1 antagonist because it has been shown to attenuate the MA-induced increase in *mPer1* expression observed in the caudate putamen [14]. This led us to hypothesize that D1 receptors might play a unique role in MA-induced resetting of clock genes and their protein products and, further, that a dopamine antagonist would block the effects of MA on these peripheral oscillators. To address this hypothesis, we subjected mice to daily, scheduled MA injections with and without pretreatment with a D1 receptor antagonist. The mice used in these experiments carried a PER2::LUC fusion protein, allowing us to monitor circadian expression of the clock gene product PER2 in tissues explanted from animals treated with MA and D1 receptor antagonist.

## Methods

### Ethics Statement

All procedures were approved by the University of Virginia Animal Care and Use Committee (protocol #2586).

### Animals

Adult, male *mPeriod2^Luciferase^* mice (PER2::LUC; originally derived from animals kindly given to us by Dr. Joseph Takahashi, University of Texas Southwestern Medical Center, Dallas, TX) were obtained from a breeding colony at the University of Virginia. These mice carry a PER2::LUC fusion protein which has been engineered to produce light when expressed in the presence of luciferin [15].

### Experimental Design

Animals were individually housed in Nalgene cages equipped with running wheels; food and water were available *ad libitum*. Activity data were recorded using ClockLab software (Actimetrics, Evanston, IL). Animals were kept on a 12∶12 light:dark (LD) cycle. Light intensity at the level of the animals’ cages was approximately 350 lux. Mice were allowed to acclimate to the housing conditions for at least 1 week prior to the start of experimental manipulations. Mice were subjected to 1 of 6 treatments: unhandled, scheduled daily saline injections, scheduled daily methamphetamine (MA) injections (2 mg/kg), a single MA injection (2 mg/kg), scheduled daily MA injections (2 mg/kg) preceded by SCH23390 (SCH, 0.05 mg/kg administered 15 min prior to MA injection), or scheduled daily SCH injections alone (0.05 mg/kg). Unhandled mice received no further manipulation until the time of sacrifice. Saline and MA-injected animals received a daily i.p. injection 7h after lights-on [zeitgeber time (ZT) 7] for 10 days (see “Drugs,” below). Mice receiving SCH pretreatment received an MA injection (2 mg/kg) 7h after lights-on, but were pretreated with an injection of SCH 15 min prior to MA injections (i.e., 6.75h after lights-on) on each day. Mice receiving SCH alone received daily injections of SCH 6.75h following lights-on for 10 days. For all groups, on the 10^th^ day of injections (or comparable experimental day for the unhandled group), approximately 1–2h prior to lights off (ZT10-11, or 3–4h post-injection), mice were sacrificed and organs harvested for tissue culture as described below. Mice receiving a single MA injection were injected i.p. with 2 mg/kg MA at ZT7 on the day of sacrifice to control for an acute effect of MA. Throughout the experimental period, unhandled mice were held in the same environmental chambers as mice receiving injections, and were therefore subjected to the same environmental noise as the injected animals.

### Locomotor Activity Analyses

**Figure 1:**
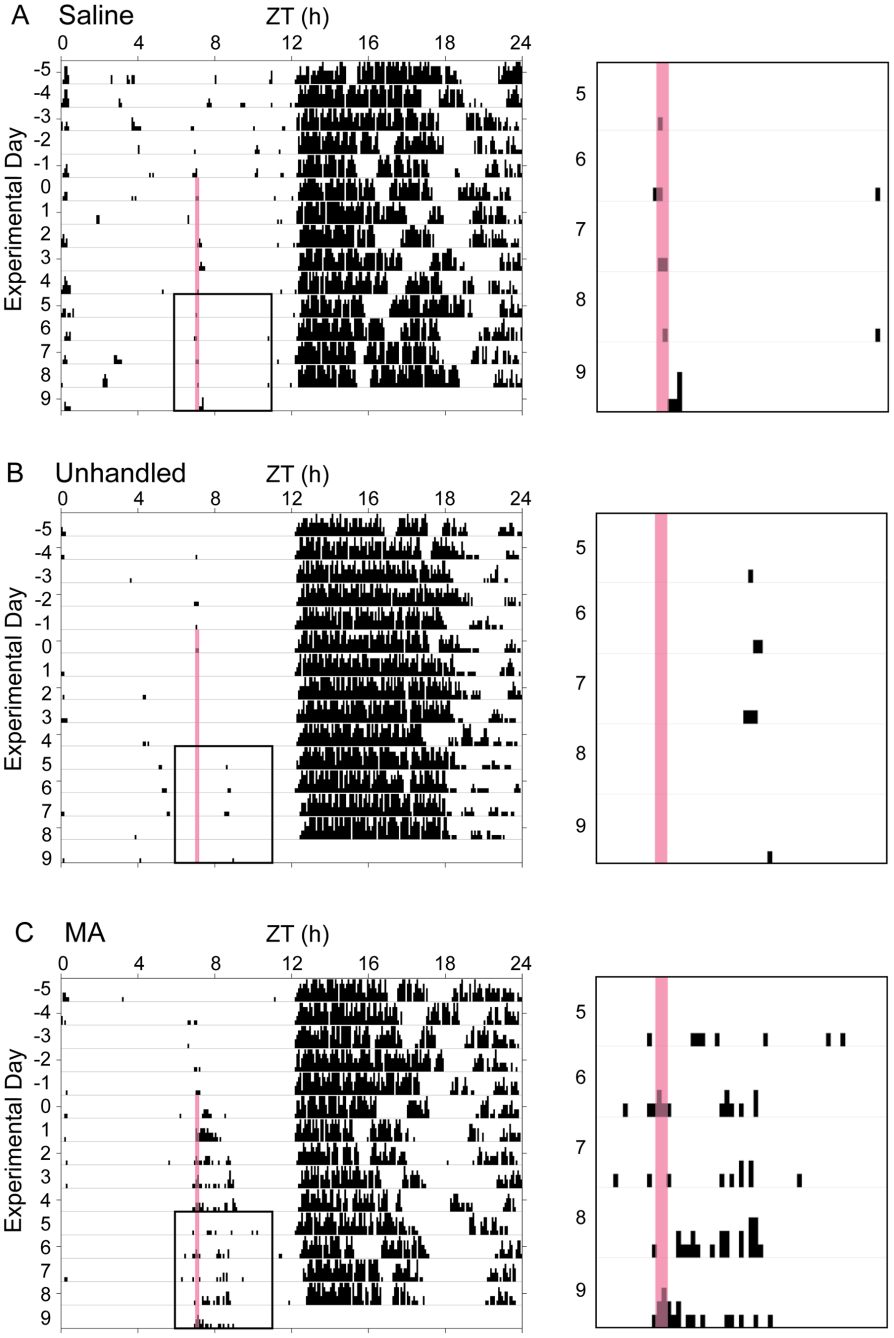
Circadian locomotor activity. Wheel running actograms from representative animals in the saline-injected (A), unhandled (B), and MA-injected (C) conditions. Mice in the injected conditions received 10 daily injections at ZT7. The pink bar indicates the time of injection (or time of environmental chamber opening in the case of unhandled mice); day 0 is the first day of injections. The left-hand panels depict daily wheel running beginning 5 days prior to the start of injections. The right-hand panels show enlarged activity data from the area outlined in the left panel (the final 5 days of injections). Methamphetamine injection resulted in an acute increase in locomotor activity with clear anticipatory activity in the minutes prior to the scheduled injection and an increase in activity following injection.

Wheel running activity of each animal was recorded and analyzed using ClockLab hardware and software (Actimetrics, Evanston, IL). Wheel running data were compressed into 5 min bins and daily activity profiles were generated in ClockLab across 4 days prior to the start of injection (excluding the day immediately preceding the injection start day, as animals were weighed on this day) and the final 7 days of the experiment (with the final day of analysis being the day of sacrifice). In cases where activity data were missing due to equipment failure, that day was excluded from the analyses for that animal. Activity levels were converted to percent of total average daily activity, and activity on days 3–9 (with injection day defined as day 0, see Fig. 1) was compared to activity on days −5 to −2 (prior to the start of the injection paradigm, or the comparable experimental day for unhandled animals). “Injection anticipatory activity” was defined as activity occurring during the hour prior to injection (ZT6-7, hour 6 to 7 post-light onset). “Injection induced activity” was defined as activity occurring in the 4 hours following the injection (ZT7-11).

The change in activity levels following the injection paradigm was not normally distributed across groups, and the standard error differed among groups. Data were therefore analyzed using a Kruskal-Wallis test followed by post-hoc Mann-Whitney U pair-wise comparisons testing for exact, 1-tailed significance.

### Drugs

Mice that received MA injections were injected with methamphetamine hydrochloride (Sigma-Aldrich, St Louis, MO) dissolved in sterile saline. Mice receiving saline injections were injected i.p. with an equivalent volume of sterile saline relative to bodyweight. SCH (SCH23390 hydrochloride; Sigma-Aldrich, St Louis, MO) was administered in sterile saline.

### Tissue Culture

Tissue culture and preparation was performed as described elsewhere [16]. Mice were anesthetized with carbon dioxide and decapitated, and tissues were harvested and placed in chilled Hanks’ balanced salt solution. Tissue was typically processed within 30 min of sacrifice. From animals in the unhandled, saline-injected, and scheduled daily MA-injected (2 mg/kg) groups, mice were sacrificed in experimental cohorts of 2 mice/day and brain, adrenal gland, cornea, liver, lung, pituitary, and salivary (submaxillary) gland were harvested [only tissues affected by MA (liver, lung, pituitary, and salivary gland) were collected from SCH-injected mice]. SCN was taken from a 300 µm coronal brain section sliced on a vibratome. The adrenal gland was hand-sliced coronally into a thin section containing both medulla and cortex. The whole cornea was removed from the eye. Anterior pituitary gland was cultured whole. The salivary gland, liver, and lung were hand sliced into thin sections. Explanted tissues were placed on Millicell culture inserts in 35-mm culture dishes in medium containing luciferin. Cultures were incubated for 6 days under constant conditions (constant darkness, constant temperature of 35°C, and no medium change). The light emitted from each individual culture dish was recorded in real-time with photomultiplier tube detectors (Hamamatsu, Bridgewater, NJ).

### Analysis PER2::LUC Bioluminescence Data

Bioluminescence data were detrended by subtracting the 24h running average from the raw data. The detrended data sets were smoothed by taking 30 min running averages. From this smoothed data set, the time corresponding to the highest level of bioluminescence that occurred on the second day in culture was considered the peak phase. Peak phases were converted into phase angles relative to the *in vivo* entraining LD cycle. Mean vectors of circular distributions based on phase of individual tissues were calculated. Rayleigh’s Uniformity test was applied to determine if there was significant clustering of the peak phases of PER2::LUC expression for each tissue (i.e., whether PER2::LUC expression for a given tissue from the same experimental group had a fixed phase relationship relative to light onset). Watson-Williams F-test was applied to evaluate the differences among treatment conditions. In two cases, tissues were lost or damaged during tissue harvesting (1 pituitary from the methamphetamine-treated group, 1 adrenal gland from the unhandled group). With the exception of salivary gland (which damps quickly and for which rhythmicity was determined by the first 36h of data only), periodogram analyses were conducted on at least 48h of luminescence data to determine the presence of a robust circadian rhythm. Tissues which failed to exhibit a significant circadian oscillation (with a period between 20 and 30h) by periodogram analysis were considered arrhythmic. Only rhythmic cultures were used for peak phase analyses. Arryhthmic tissues from each treatment condition are noted in the text and in Table S1. Periodogram amplitude data (for all tissues except salivary gland) were also collected from at least 48h of luminescence.

## Results

### Locomotor Activity

**Figure 2:**
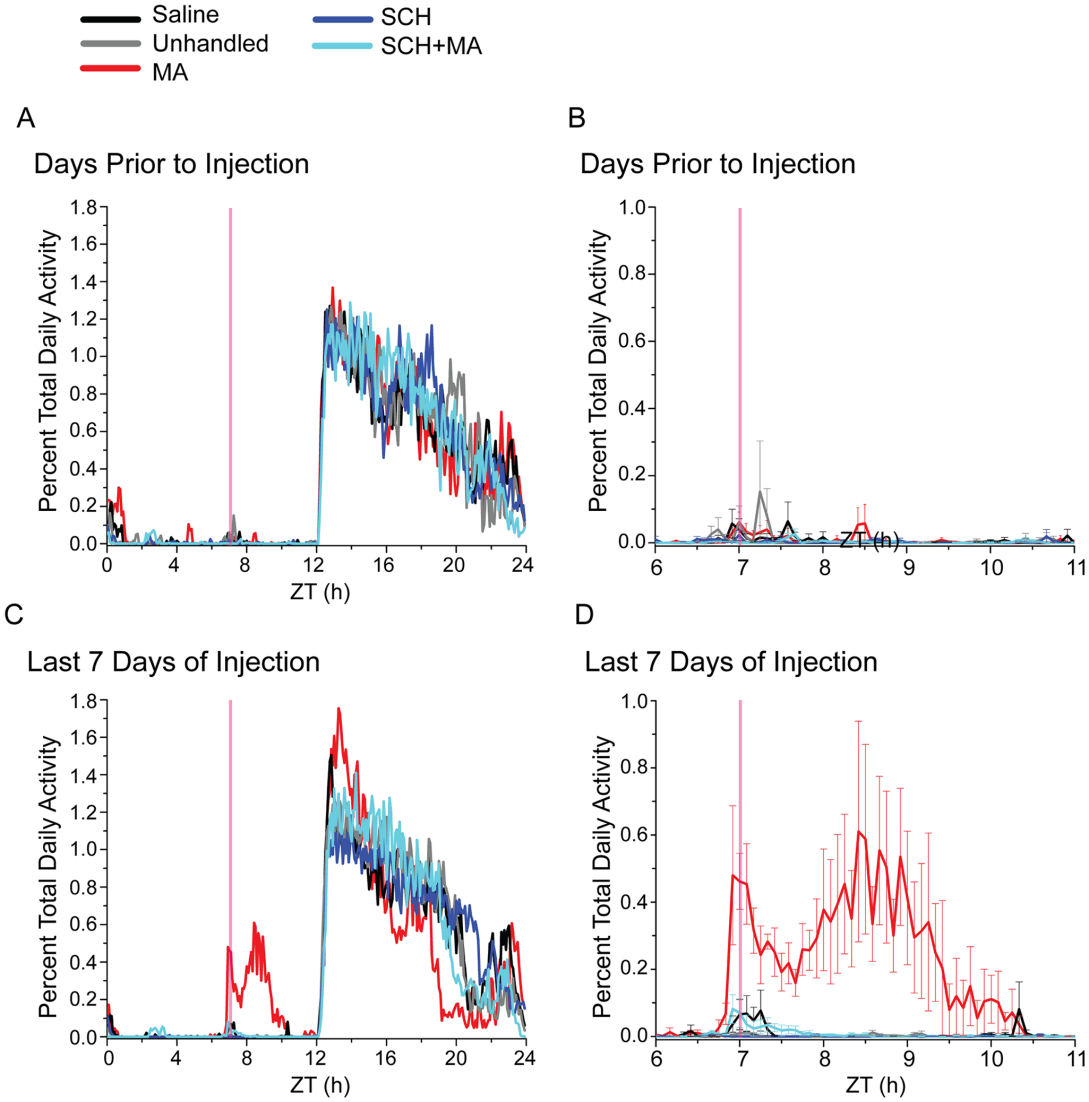
Average activity profiles from mice receiving scheduled daily saline (black), left unhandled (gray), receiving scheduled daily MA (2 mg/kg) injections (MA, red), receiving daily D1 receptor antagonist injections (SCH, blue), and receiving pretreatment with a D1 receptor antagonist followed by MA (SCH+MA, aqua). Activity data are averaged from 5 min bins across days 4 days prior to the start of injections (day −5 to −2, where day 0 is the first day of injections; Fig. 2A and 2B) or the last 7 days of injections (day 3 to 9; Fig. 2C and 2D). Data are expressed as percent of total daily activity, and zeitgeber time (ZT) is along the x-axis. The time of MA injection (ZT7) is indicated by the pink vertical line. A) and B) illustrate the full 24h activity profiles, plotted as the mean percent of total daily activity. C) and D) are enlarged data from ZT6-11, plotted as the mean ± SEM percent of total daily activity. Note the robust increase in activity level beginning immediately prior to and extending for 3–4h hours following injection in the MA group (D).

**Figure 3:**
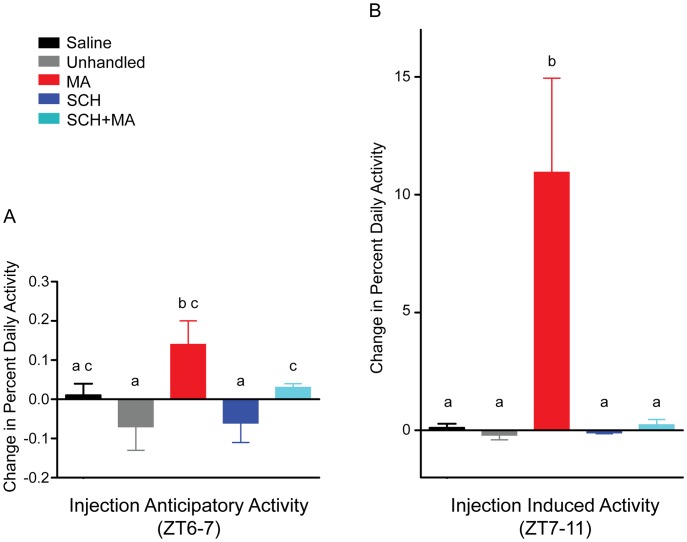
Scheduled daily MA injections result in anticipatory and induced activity. A) Injection anticipatory activity. For each mouse, activity level from ZT6-7 (1h prior to injection) was scored (as percent of average daily activity) on the last 7 days of injection and compared to that animal’s activity level from ZT6-7 on 4 days prior to the start of the injection paradigm (ZT6-7 corresponds to the area to the left of the pink vertical bar in Fig. 2B, D). The difference in activity levels are presented as mean ± SEM. Different letters above bars represent significant differences (*p*<0.05) between groups (i.e., “a c” differs from “b c” but not from bars designated as “a” or “c” alone, “b” differs from “a c” and “a,” “c” differs from bars labeled “a” alone). B) Injection induced activity. For each mouse, activity level from ZT7-11 (4h post injection) was scored (as percent of average daily activity) on the last 7 days of injection and compared to the activity level from ZT7-11 on 4 days prior to the start of the injection paradigm (ZT7-11 corresponds to the area to the right of the pink vertical bar in Fig. 2B, D). The difference in activity levels are presented as mean ± SEM. Different letters above bars represent significant differences (*p*<0.05).

Repeated, scheduled injections of MA (at ZT7, in the middle of the light portion of the LD cycle) resulted in anticipatory activity (Fig. 1–3). Mice receiving MA demonstrated an increase in the percentage of their daily activity occurring from ZT6-7 as a result of the scheduled injections (*p*<0.05 compared to saline-injected and unhandled controls; Fig. 3A). Pretreatment with SCH (15 min prior to MA injection) did not significantly suppress methamphetamine-anticipatory activity, but resulted in levels intermediate to the control and MA-treated mice (Fig. 3A, Fig. S1). Scheduled daily injections of saline or SCH alone (at ZT7) did not result in anticipatory activity (Fig. 3A, Fig. S1).

Not surprisingly, increased activity levels were also observed during the 4h period following MA injection (“injection-induced activity, ” Fig. 1–3). MA-injection resulted in an increase in the percent of daily activity occurring from ZT7-11 (4h post-injection), as compared to all other treatment conditions (*p*<0.05; Fig. 3B). This activity increase was suppressed by SCH pretreatment.

The overall activity profile of MA-treated mice did not differ greatly from undhandled or saline-injected animals. However, an increase in overall activity was observed in the early night, with a smaller percentage of daily activity occurring in the late night (Fig. 2C). Unhandled animals, saline-injected animals, and animals that received only the D1 receptor antagonist SCH 23390 (SCH) had normal circadian activity profiles, with high levels of wheel-running during the dark portion of the LD cycle and low levels during the light (Fig. 1–2, Fig. S1).

### Phase of PER2::LUC Expression in Peripheral Tissues

The impact of scheduled MA injections on behavior led us to hypothesize that circadian rhythms within individual tissues would also be influenced by MA. To test this hypothesis, we examined the impact of scheduled, daily injections of MA and a D1 receptor antagonist on the phases of several tissues relative to the LD cycle and on synchrony among them, as measured by peak PER2::LUC expression. Here we define synchrony as consistency in the phase of a particular tissue from animals in a given treatment group.

#### Effects of Scheduled MA Injections

**Figure 4:**
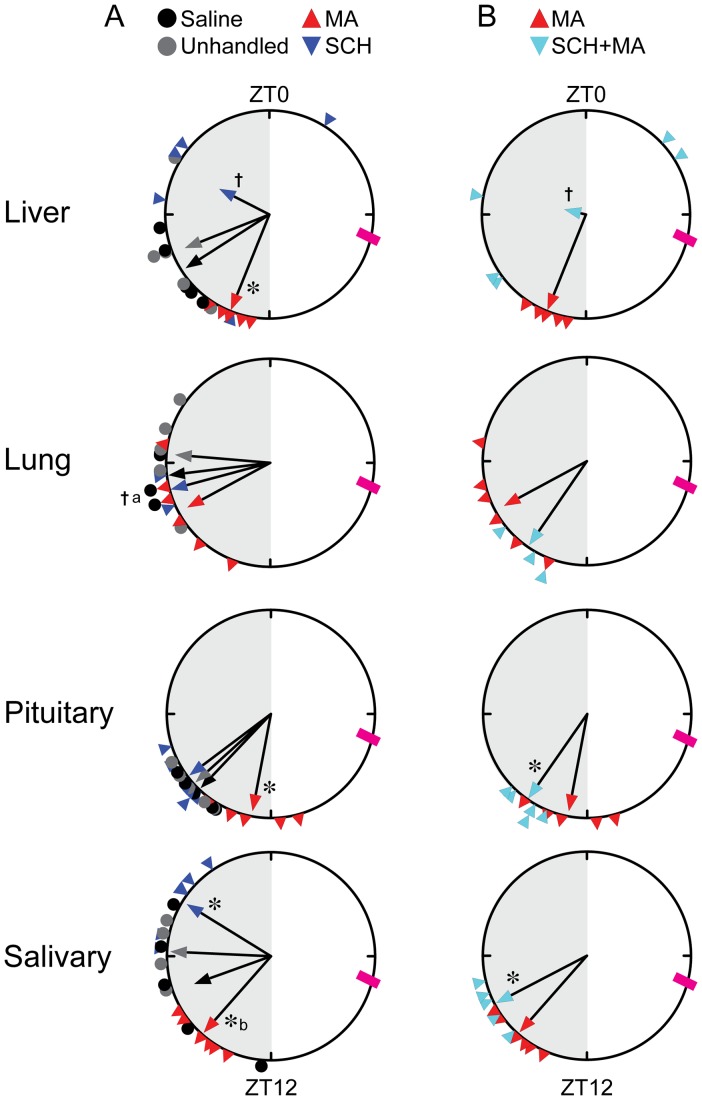
MA and SCH injections affect the phase of PER2::LUC expression in circadian oscillators. Rayleigh plots of the phases of peak PER2::LUC expression in liver, lung, pituitary gland, and salivary gland. Rayleigh plots can be read as a 24h clock, with ZT0 at the top and ZT12 at the bottom of the circle. Arrows represent the average peak phase of PER2::LUC expression (mean vectors for the circular distributions) of each group. The length of the vector represents the strength of the phase clustering while the angle of the vector represents the mean phase. Individual data points are plotted outside the circle. The pink box at ZT7 indicates the timing of MA injection for MA-treated groups. A) Data from groups that were saline-injected, unhandled, injected with MA (MA), or injected with SCH23390 alone (SCH). MA resulted in a significant phase advance in liver and pituitary gland (*, *p*<0.05 compared to saline, unhandled, and SCH groups). In salivary gland, MA advanced phase relative to unhandled controls (*b, *p*<0.05) whereas SCH significantly delayed phase compared to all other groups (saline, unhandled, or MA-injected). SCH administration resulted in desynchrony among livers (†, Rayleigh *p*>0.10). SCH administration also resulted in arrhythmicity in 4/6 lungs. Lungs from SCH-treated animals also failed to demonstrate significant synchrony (†a, Rayleigh *p*>0.10), although this was probably the result of decreased statistical power due to the low number of remaining rhythmic cultures. B) Data from groups that were pretreated with SCH before MA injection (SCH+MA) or injected with MA alone (same data as A). SCH pretreatment significantly attenuated the MA-induced phase shift in pituitary and salivary gland (*, *p*<0.05) and disrupted synchrony among livers (†, Rayleigh *p*>0.10). SCH+MA also resulted in arrhythmicity in 2/5 lungs.

**Figure 5:**
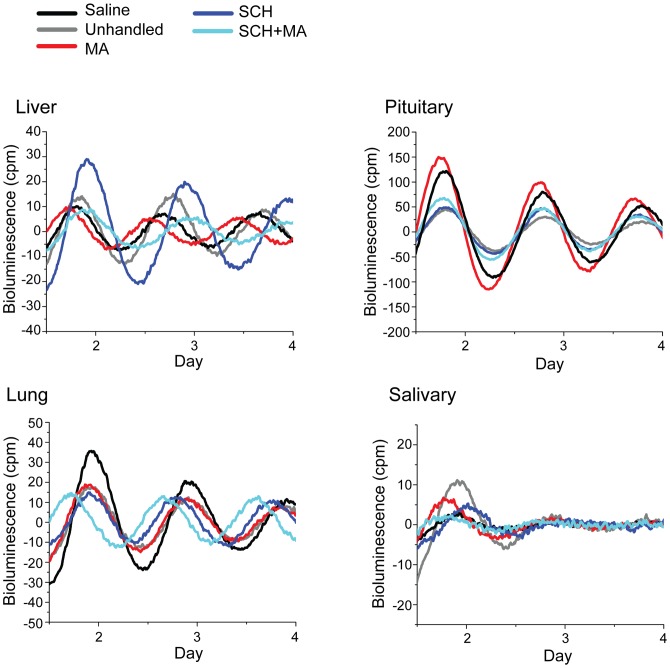
Representative PER2::LUC traces from liver, lung, pituitary, and salivary gland. Traces depict baseline subtracted, detrended bioluminescence (PER2::LUC) from mice in the saline-injected (black), unhandled (gray), MA-injected (red), SCH-injected (blue), and SCH-pretreated (SCH+MA, aqua) conditions.

Scheduled MA injection altered the phase of some, but not all, peripheral tissues. Ten days of scheduled MA injections (2 mg/kg at ZT7) significantly advanced the phase of PER2::LUC expression in liver and pituitary gland (compared to unhandled and saline-injected controls, *p*<0.05; Fig. 4–5, Table 1). Salivary glands from MA-treated animals were advanced compared to unhandled controls (p<0.05), but did not differ significantly from the saline-treated group. There was also a trend towards an advance of the phase of lung compared to unhandled controls, but this did not reach significance (*p* = 0.075; Fig. 4A). Other tissues tested (adrenal, cornea, SCN) were not affected by the scheduled injections (Fig. S2). A single dose of methamphetamine on the day of tissue collection did not alter the phase of any tissues, demonstrating that the effects of MA on circadian phase are not due to an acute response to the drug (data not shown). Scheduled daily saline injections had very little effect on PER2::LUC rhythmicity, with no observed difference in the phase of tissues from saline-injected animals compared to unhandled controls. None of the treatment conditions significantly affected the periodogram amplitude of the SCN or the peripheral oscillators, suggesting no change in the robustness of the oscillators.

**Table 1:** 

Condition	Liver			Lung			Pituitary			Salivary		
**Saline**	15.82±0.51			17.54±0.30			14.90±0.32			16.68±1.19		
**Unhandled**	16.56±0.89			18.32±0.71			15.19±0.36			18.18±0.48		
**MA**	13.46±0.25			16.11±0.70			12.71±0.55			14.76±0.33		
**SCH**	19.81±1.89			17.00±0.39			15.54±0.30			20.11±0.49		
**SCH+MA**	18.89±3.01			14.30±0.49			14.32±0.26			16.17±0.33		

Mean ± SEM (circulaer) peak PER2::LUC phase, presented in ZT (in h, where ZT0 is lights-on and ZT7 is the time g004of MA injection). Numbers of tissues scored are presented in Table S1. See Fig. 4 for graphical representation and statistics.

#### Effects of D1 Receptor Antagonist

Daily injections of the D1 receptor antagonist SCH 23390 affected phase synchrony in peripheral oscillators. Ten days of injections with SCH alone decreased phase synchrony among livers and lungs (Rayleigh’s Uniformity test, *p*>0.10; Fig. 4A). SCH injection also affected rhythmicity in lungs, with 4/6 lungs from the SCH-injected group failing to meet the defined criteria for rhythmicity (Table S1). SCH-injection resulted in a delayed phase in salivary glands (*p*<0.05; Fig. 4–5, Table 1), but had no effect on the phase of pituitary glands. SCH injection did not affect the periodogram amplitude of any of the rhythmic tissues.

#### Combinatorial Effects of MA and a D1 Receptor Antagonist

We next tested the ability of SCH 23390 to block MA-induced shifts in the phase of peripheral tissues. Mice were given SCH 15 min prior to a scheduled MA injection for 10 days (“SCH+MA”). SCH attenuated the MA-induced phase shift of PER2::LUC expression observed in pituitary and salivary gland (SCH+MA v. MA alone, *p*<0.05 for both tissues; Fig. 4–5, Table 1). SCH pretreatment (followed by MA) resulted in arrhythmicity in 2/5 lungs (Table S1) and decreased phase synchrony among livers (Rayleigh’s Uniformity test, *p*>0.10; Fig. 4B). SCH pretreatment did not affect the periodogram amplitude of rhythmic tissues.

## Discussion

### Effects of Scheduled MA on Circadian Rhythms

Daily injections of MA in the mid subjective day result in anticipatory and induced locomotor activity. Scheduled MA injections also shift the phase of peak PER2::LUC expression in liver, pituitary, and salivary gland toward the time of the injection. This confirms previous work demonstrating that MA can affect the phase of liver [13], and provides the first demonstration that pituitary and salivary gland rhythms are affected by repeated MA injections. Scheduled saline-injections had no effect on the phase of any peripheral tissues, nor did a single, acute dose of MA at the same time of day. Thus, the effect of scheduled, daily MA-injections on peripheral circadian rhythms must be due to a longer-term, MA-dependent change in circadian signaling or entrainment, which develops over time.

There was no change in the phase of the SCN following scheduled daily MA injections. The anticipatory rhythms of locomotor activity may therefore be due to an effect of MA on an extra-SCN brain oscillator. MA-injections may be stimulating the so-called “methamphetamine sensitive circadian oscillator (MASCO),” a circadian pacemaker which has been shown to influence circadian rhythms in the absence of the SCN [7], [9].

Likewise, the resistance of the SCN to MA suggests that the effect of MA on rhythms in the periphery is unlikely to rely upon SCN-driven mechanisms. The shift in peripheral rhythms may be due to effects on local clocks and/or be secondary to the effect of the injections on locomotor activity (and, presumably, body temperature and feeding). Scheduled injections of MA produce both modest increases of anticipatory activity (activity prior to the time of scheduled injection) and large levels of injection-induced (post-injection) activity. This altered locomotor activity could feed back into the circadian system to influence clock mechanisms upstream of or within peripheral tissues.

### A Role for Dopamine 1 Receptors in Peripheral Rhythms

Scheduled daily injections of a D1 receptor antagonist (SCH) disrupted phase synchrony among livers and lungs and delayed the phase of peak PER2::LUC expression in salivary glands. Moreover, SCH injections alone and prior to MA resulted in arryhthmicity in several lung explants. For some tissues dopamine signaling (possibly far upstream from the tissue itself) appears to contribute to the maintenance of normal circadian entrainment and, in the case of lung, rhythmicity itself. Of the extra-SCN tissues influenced by MA injections, pituitary gland is the only one that has been demonstrated to contain dopamine receptors [17] and, surprisingly, it was not affected by the daily SCH injection. This suggests that the effects of dopaminergic signaling on rhythms within peripheral tissues are occurring indirectly, most probably through effects in brain. The striatum is a good candidate for a neural oscillator that could respond to dopamine signaling, as the effects of MA itself may be due, at least in part, to changes in rhythmicity/clock gene expression observed within this region [11], [13], [14]. It will be interesting to test the hypothesis that the actions of dopamine on peripheral oscillators are occurring as a result of changes in clock function within the striatum or another neural locus. Another possibility is that SCH is influencing peripheral oscillators via signaling from other tissues which do contain D1-like receptors such as the adrenal gland [18], [19] or the intestine [20]. It is possible that dopaminergic-signaling within these tissues mediate the response of peripheral clocks to MA as well (although in this study the adrenal did not shift in response to scheduled MA). The finding that SCH can alter circadian rhythms in peripheral tissues is intriguing, but provides only an initial glimpse into the role of dopamine in control of peripheral circadian clocks. Further work testing the role of D2 receptors and dopaminergic pathways into the circadian clock mechanism are necessary before we can begin to understand the role of dopamine in peripheral clock function.

### Dopamine Receptors Regulate MA-induced Phase-Shifts

SCH pretreatment suppressed MA-induced locomotor activity and attenuated (but did not fully suppress) MA-anticipatory activity. Pretreatment with SCH before daily MA injections resulted in complex circadian outcomes in peripheral tissues. SCH pretreatment followed by MA injection disrupted rhythmicity in livers and lungs. This finding further supports the conclusion that dopaminergic signaling may be necessary for proper entrainment in these tissues (as discussed above). In pituitary and salivary glands SCH pretreatment partially blocked MA-induced phase shifts. In the salivary glands, this may have been due to an additive effect of MA and SCH, as MA injections advanced and SCH injected alone delayed the phase, resulting in a zero sum effect. Alternatively, there may be a more mechanistic role for D1-signalling in MA-induced phase-shifts. Testing the effect of scheduled daily MA injections at different times of day or examining the dose response to SCH may provide further insight. As SCH alone had no effect on pituitary gland, the ability of SCH to attenuate MA-induced phase shifts in this tissue is likely due to a more direct role of D1 receptors in mediating the actions of MA within the pituitary oscillator. However, the effect of SCH on pituitary could be far downstream of the receptor itself, as SCH also suppressed the locomotor stimulation observed following MA injections and the change in pituitary could be a consequence of the change in locomotor activity or subsequent feeding/body temperature changes.

Unraveling the pathways linking dopamine-receptor activation and the clock will shed light on the mechanism by which methamphetamine can influence circadian rhythms of gene expression and behavior. Toward this end, further exploration of the roles of D2 and NMDA receptors in MA-influenced circadian outcomes is necessary. Scheduled, daily injections of either a D1 (SKF38363) or D2 receptor (quinpirole) agonist have been reported to result in increased activity at the time of injection on the withdrawal day, suggesting that activation of either D1 or D2 receptors alone is sufficient to result in persistence of increased, injection-related activity [12]. Interestingly, while D1 receptor antagonists block MA-induced changes in clock gene/protein expression in the caudate putamen [14], as well as pituitary and salivary glands (as demonstrated in the present study), the D2 receptor antagonist sulpiride has been shown to facilitate the MA-induced increase in *Per1* in the caudate [14]. These results may be due to the opposing actions of D1 and D2-receptors on intercellular signaling pathways [21]. The balance of D1 and D2-receptor activation may mediate the impact of MA on circadian rhythms in a tissue-specific fashion. D1 modulation of D2 receptor action may explain the disrupted rhythms we observed in the livers and lungs from MA-injected mice pretreated with SCH. Interestingly, there is also evidence that D2 receptor (although not D1 receptor) signaling may be important for normal patterns of PER2 expression within the striatum [22].

The interaction of D1, D2, and NMDA receptors in MA-induced changes in circadian activity may represent changes in receptor levels and/or sensitization of the response to MA over time. Repeated injection of MA can lead to sensitization of both locomotor activity and *mPer1* expression in the caudate [14]. It has been suggested that behavioral sensitization may underlie the effects of MA on circadian rhythms [12]. Additionally, or alternatively, it is possible that alterations in circadian gene expression are necessary for general behavioral sensitization to repeated psychostimulant exposure. Indeed, mutations in clock genes result in abnormal sensitization to cocaine in both drosophila [23] and mice [24], [25].

### Conclusion

The way in which circadian rhythms are generated and maintained in the periphery is poorly understood. The limited available data suggest roles for the central pacemaker in the SCN, the as yet unidentified food entrainable and methamphetamine sensitive oscillators, as well as local signaling mechanisms in setting the clock in organs throughout the body. There is a strong influence of feeding time and metabolic signals on these peripheral oscillators, making them particularly sensitive to exogenous challenges. Our data demonstrate a role for D1-receptor signaling in both the robustness and phase of rhythms in some peripheral tissues. This opens the door for further research investigating the impact of the dopaminergic system on the maintenance of synchrony within the circadian system. The present study showed that scheduled MA treatment is capable of shifting the phase of peripheral tissues, an effect that, in salivary and pituitary glands, is achieved at least in part through D1-receptor signaling. The failure of SCH administration to block MA-induced phase-shifts in liver demonstrates that there are other pathways through which MA can influence circadian rhythms in the periphery.

This research highlights the importance of understanding the impact of drugs of abuse and therapeutic pharmaceutical agents on circadian rhythms. The impact of compounds such as MA on individual tissues and the synchrony of the circadian system as a whole could have profound consequences for the efficacy and physiological consequences of using and/or abusing this and other drugs.

## Supporting Information

Figure S1 **Circadian locomotor activity. Wheel running actograms from representative animals in the SCH (A) and SCH +MA (B) conditions.** Mice received 10 daily injections (SCH administered at ZT6.75, MA administered at ZT7). The pink bar indicates ZT7 (the time of MA injection). Day 0 is the first day of injections. The left-hand panels depict daily wheel running beginning 5 days prior to the start of injections. The right-hand panels show enlarged activity data from the area outlined in the left panel (the final 5 days of injections).
(TIF)

Figure S2 **Representative PER2::LUC traces from SCN, cornea, and adrenal gland.** Traces depict baseline subtracted, detrended bioluminescence (PER2::LUC) from mice in the saline-injected (black), unhandled (gray), and MA-injected (red) conditions. Scheduled, daily MA-injections had no effect on the phase of any of these tissues.
(TIF)

Table S1 **Number of animals/tissues scored for each condition.** Number in parentheses indicates the number of tissues that failed to meet the pre-determined criteria for rhythmicity. * indicates 1 tissue from this group was lost due to damage during harvesting.
(DOCX)
