# Supplementary figures and images for: Methamphetamine and Dopamine Receptor D1 Regulate Entrainment of Murine Circadian Oscillators
Source: PLoS One. 2013 Apr 23;8(4):e62463. doi: 10.1371/journal.pone.0062463 (PMC3633847; doi:10.1371/journal.pone.0062463)

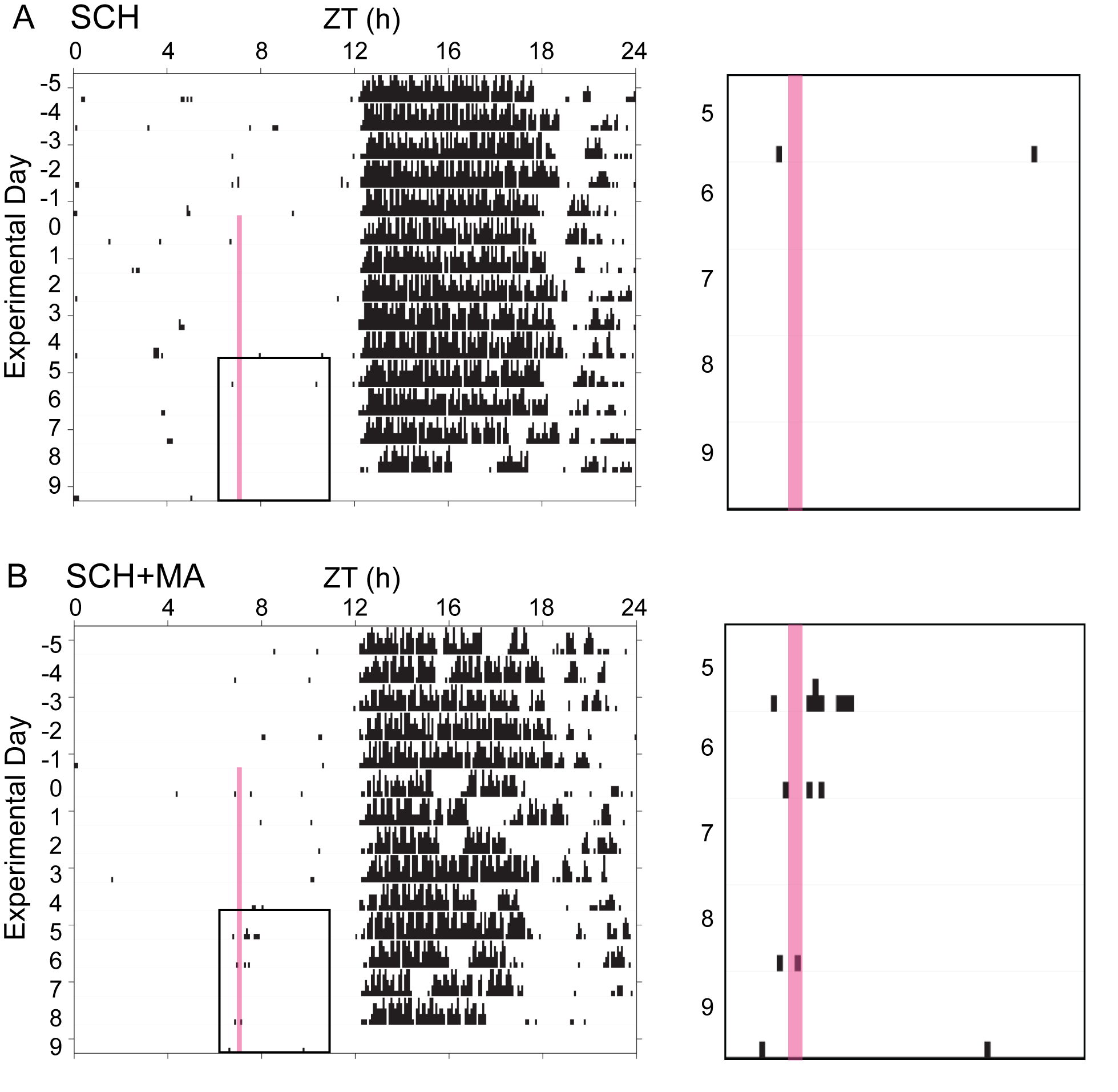

Supplement: Figure S1 — Circadian locomotor activity. Wheel running actograms from representative animals in the SCH (A) and SCH +MA (B) conditions. Mice received 10 daily injections (SCH administered at ZT6.75, MA administered at ZT7). The pink bar indicates ZT7 (the time of MA injection). Day 0 is the first day of injections. The left-hand panels depict daily wheel running beginning 5 days prior to the start of injections. The right-hand panels show enlarged activity data from the area outlined in the left panel (the final 5 days of injections). (TIF) [file pone.0062463.s001.tif]

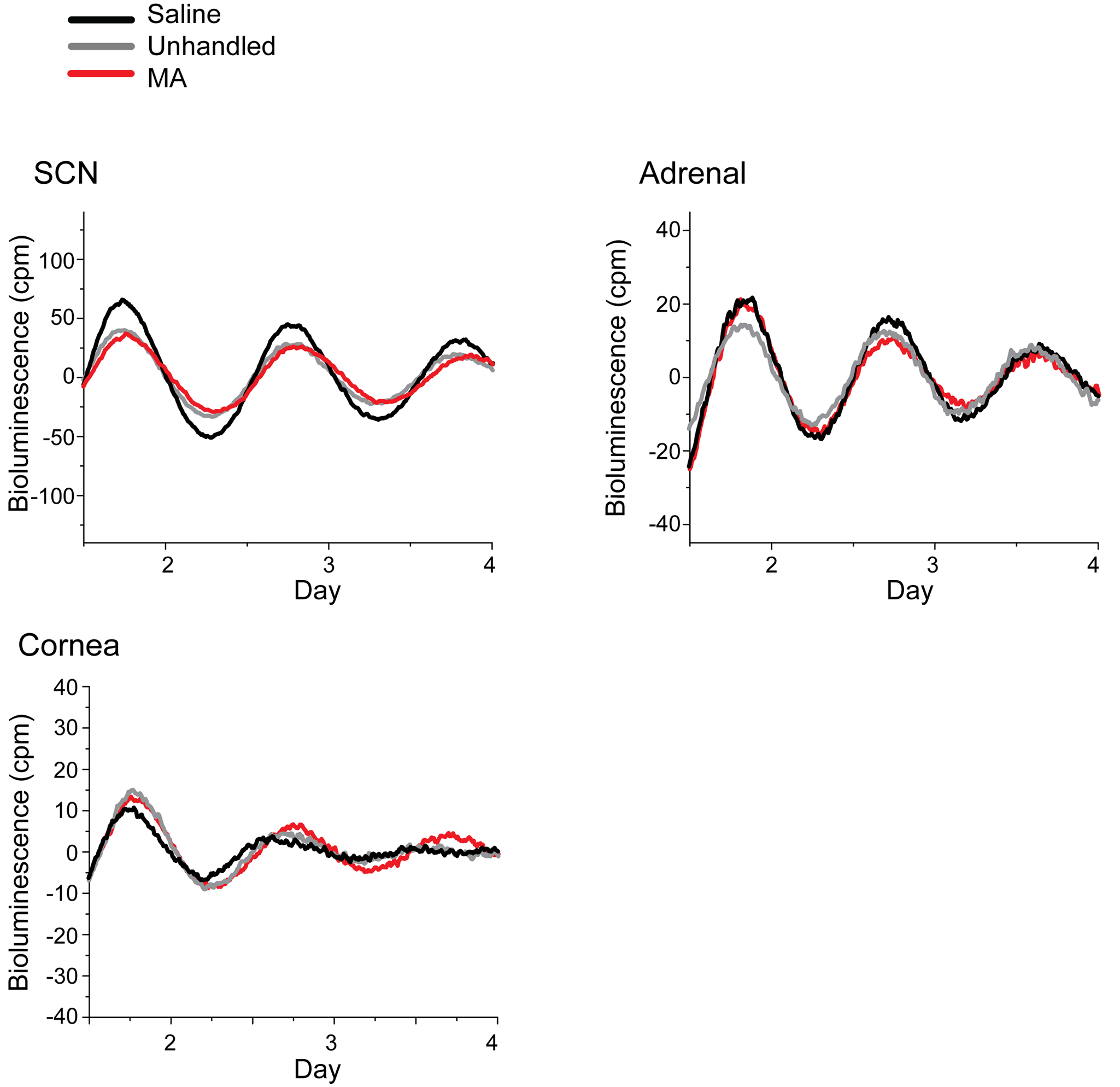

Supplement: Figure S2 — Representative PER2::LUC traces from SCN, cornea, and adrenal gland. Traces depict baseline subtracted, detrended bioluminescence (PER2::LUC) from mice in the saline-injected (black), unhandled (gray), and MA-injected (red) conditions. Scheduled, daily MA-injections had no effect on the phase of any of these tissues. (TIF) [file pone.0062463.s002.tif]
