# Supplementary material for: Methamphetamine and Dopamine Receptor D1 Regulate Entrainment of Murine Circadian Oscillators
Source: PLoS One. 2013 Apr 23;8(4):e62463. doi: 10.1371/journal.pone.0062463 (PMC3633847; doi:10.1371/journal.pone.0062463)
Supplement: Table S1 — Number of animals/tissues scored for each condition. Number in parentheses indicates the number of tissues that failed to meet the pre-determined criteria for rhythmicity. * indicates 1 tissue from this group was lost due to damage during harvesting. (DOCX) [file pone.0062463.s003.docx]

Table S1.

| **Condition** | **Animals (Activity)** | **SCN** | **Adrenal** | **Cornea** | **Liver** | **Lung** | **Pituitary** | **Salivary** |
| --- | --- | --- | --- | --- | --- | --- | --- | --- |
| **Saline** | 5 | 5 | 4 (1) | 5 | 5 | 5 | 5 | 5 |
| **Unhandled** | 5 | 5 | 4* | 5 | 5 | 5 | 5 | 4 (1) |
| **MA** | 6 | 6 | 6 | 6 | 5 (1) | 6 | 5* | 6 |
| **SCH** | 6 |  |  |  | 5 (1) | 2 (4) | 6 | 6 |
| **SCH+MA** | 5 |  |  |  | 5 | 3 (2) | 5 | 5 |
